# Gastric cancer adapts high lipid microenvironment via suppressing PPARG-FABP1 axis after arriving in the lymph node

**DOI:** 10.1016/j.redox.2025.103759

**Published:** 2025-07-17

**Authors:** Yinan Liu, Liang Tang, Baifu Peng, Shaoji Zhao, Ziling Shao, Kaiyu Sun, Jinning Ye, Wei Chen, Jianbo Xu

**Affiliations:** a Department of Gastrointestinal Surgery, The First Affiliated Hospital of Sun Yat-sen University, No. 58, Zhongshan 2 Road, Guangzhou, Guangdong, 510080, People's Republic of China; b Department of General Surgery, Shengjing Hospital of China Medical University, No.36, Sanhao Street, Heping District, Shenyang, 110000, Liaoning Province, People's Republic of China; c Department of Hepatobiliary Surgery, The First Affiliated Hospital of Sun Yat-sen University, No. 58, Zhongshan 2 Road, Guangzhou, Guangdong, 510080, People's Republic of China

**Keywords:** Gastric cancer, Arachidonic acid, Ferroptosis, Lymphatic channels

## Abstract

**Aim:**

Gastric cancer (GC) primarily metastasizes through lymphatic channels, although lymphatic metastasis remains relatively inefficient. Changes in cellular metabolism, known as metabolic reprogramming, plays a significant role in the adaptive survival of cells during the process. Therefore, understanding the mechanism underlying metabolic reprogramming in lymph node (LN) metastasis is crucial for the development of targeted therapies for advanced gastric cancer. This study aimed to investigate the metabolic adaptations of GC cells during LN metastasis, with a particular focus on lipid metabolism reprogramming.

**Methods:**

Non-targeted lipidomic sequencing, combined with tumor cell flow sorting and RNA sequencing, was used to explore differences in lipid microenvironments and changes in lipid metabolism pathways between lymph nodes and primary tumors. Single-cell sequencing data were analyzed to confirm these results. Transmission electron microscopy, BODIPY 581/591 staining, and ferroptosis inhibitors were used to confirm the effects of arachidonic acid (AA) on ferroptosis sensitivity in gastric cancer. Public databases and ChIP-qPCR tests were used to investigate the role of PPARγ pathway in regulating FABP1 transcription.

**Results:**

Lipid metabolism pathway was inhibited following lymph node metastasis, with reduced lipid catabolites observed in the lymph nodes. Single-cell data also supported these findings. Physiological concentrations of AA were shown to increase ferroptosis sensitivity, lipid peroxidation, and mitochondrial damage in gastric cancer cells. FABP1 was significantly downregulated in lymph nodes, which mediated the uptake of AA, mitochondrial destruction, and lipid peroxidation. Further analysis revealed that PPARγ, a regulator of FABP1 transcription, was significantly downregulated after lymph node metastasis. Furthermore, our findings revealed that AA reduced the stability of PPARγ protein.

**Conclusion:**

The high concentration of AA in the lymph nodes microenvironment can increase the sensitivity of gastric cancer cells to ferroptosis. Mechanically, AA inhibits the PPARγ pathway to downregulate FABP1 expression, thereby suppressing AA uptake and preventing ferroptosis of gastric cancer cells.

## Highlights

• PPARG pathway directly promotes FABP1 transcription in GC cells.
• Arachidonic acid suppresses PPARG expression in GC cells in a dose-dependent manner.
• High concentrations of Arachidonic acid in LNs suppressed the PPARG pathway.

## 1 Introduction

Gastric cancer (GC) is one of the most prevalent malignant tumors worldwide, accounting for approximately 0.75 million deaths in 2020 [1]. In China, the majority of new cases are diagnosed at advanced stages, often accompanied by lymph node (LN) metastasis, which is associated with poor long-term outcomes [2]. Recent studies have reported that LN metastatic tumor cells are potential sources of organ metastasis and recurrence, further complicating chemotherapy and immunotherapy efforts [[3], [4], [5]]. However, LN metastasis is an inherently inefficient process in which most cells undergo anoikis, immune elimination, and energy deficiency, leading to cell death after leaving the primary tumor site [6]. Thus, exploring the molecular mechanisms that enable the survival of these rare, resistant cells is important for advancing therapeutic strategies.

Metabolic reprogramming occurs during oncogenesis and tumor progression and profoundly influences tumor growth, cell death, and immune tolerance [7,8]. Previous studies have primarily focused on the discrepancies between tumors and normal tissues, such as the Warburg effect [9]. However, in recent years, an increasing number of studies have emphasized the role of metabolic reprogramming during tumor progression. Disseminated cancer cells readjust their metabolic profile to adapt to the new microenvironment of the colonized site [10,11]. Among these alterations, tumors often undergo lipid metabolism reprogramming to meet lipid demands and cell death stress [12]. Mukherjee et al. reported that adipocyte-induced ovarian cancer lipid metabolism reprogramming in lipid enriched microenvironment promotes tumor metastasis and platinum resistance [13]. Similarly, other studies have also demonstrated that the elevation of lipid oxidation promotes tumor metastasis [[14], [15], [16]]. In contrast, increased fatty acids (FA) orchestrate oxidative stress by modulating cell death, which may impede tumor progression [[17], [18], [19]]. Different fatty acids exert diverse effects on cell death. Li et al. reported that fatty acid beta-oxidation (FAO) increased mitochondrial membrane lipids, protecting cells from apoptosis [20]. however, FAO induced cell death under chemotherapy treatment [21]. Polyunsaturated fatty acid (PUFA) accumulation is the key process in ferroptosis, whereas monounsaturated fatty acid (MUFA), oleic acid, prevents ferroptosis of cancer cells in the tumor draining lymph [4]. Therefore, it is important to describe the effects of various fatty acids on GC cells to understand the mechanisms of lipid reprogramming in LN metastasis.

Fatty acid-binding proteins (FABPs), a family of lipid chaperone proteins including FABP1-9, plays critical roles in regulating fatty acid uptake, lipid transportation, and storage [22]. Each FABPs has a unique systemic distribution in normal tissues and specific functions [23]. Nevertheless, evidence suggests that FABPs are also encoded in tumor tissues and regulate lipid metabolism to regulate cancer progression. For instance, increased FABP4 expression in colon [24], breast [25]and pancreatic cancer [26]promotes tumor progression by altering lipid metabolism. FABP1, originally identified in the liver tissues as a binder of fatty and bile acids [27]. Qiu et al. reported the prognostic function of FABP1 in gallbladder cancer with hepatic invasion [28]. Furthermore, single-cell sequencing (sc-seq) revealed that FABP1 induces an immunosuppressive microenvironment in liver cancer [29]. PPARα-FABP1 axis regulated intestinal fatty acid uptake, promoting nonalcoholic steatohepatitis [30]. While some studies have suggested that FABP1 is abnormally increased in advanced stages and plays a role as a biomarker in gastric cancer [31,32], it functional role and underlying molecular mechanisms in GC progression remain poorly understood.

Therefore, this study aimed to describe the discrepancies in lipid metabolic profile between the primary site and metastatic LN in GC, as well as explore lipid metabolism reprogramming in GC cells following dissemination to draining LNs. Additionally, the study sought to explore the effect of the LN microenvironment on GC cells and the underlying signaling pathways of lipid metabolism alterations for microenvironment adaptation.

## 2 Materials and methods

### 2.1 Patients and tissue specimens

Tumor and paired para-carcinoma tissues were collected from patients diagnosed with gastric cancer at the First Affiliated Hospital of Sun Yat-sen University (Guangzhou, Guangdong, China). All patients underwent radical excision. All tissue specimens were separated and frozen at −80 °C. Ethical approval for research on human subjects was obtained from the Institutional Review Board of the First Affiliated Hospital of Sun Yat-Sen University. Each participant signed an informed consent form before participating in the study. The human study was performed in accordance with Helsinki Declaration.

### 2.2 Cell lines, cell culture, and transfection

Human gastric cancer cell lines (AGS, MKN45 and HGC27) were purchased from the Chinese Academy of Sciences Shanghai Branch Cell Bank (Shanghai, China). AGS cells were cultured in DMEM/F12 (Gibco, Thermo Fisher Scientific, Inc., Waltham, MA, USA) and MKN45 and HGC27 cells were cultured in DMEM (Gibco) at 37 °C with 5 % CO_2_. All culture medium was supplemented with 10 % foetal bovine serum (FBS; Biological Industries, Israel) and 1 % antibiotics (penicillin/streptomycin, Gibco). The cell lines were tested for potential mycoplasma contamination and confirmed mycoplasma-negative.

Drug concentrations: Fer-1 (10 μM), Z-VAD (5 μM), Nec-1 (1 μM), RSL3 (Cell line-specific concentrations: 5 μM for MKN45; 2.5 μM for AGS; 1uM for HGC27). Briefly, the cells were grown on 6 well culture plates with 10 % FBS medium 24 h before treatment. Then, Rsl-3 was added to the medium for 12–24 h.

According to the manufacturer's instructions, siRNA transfection was performed using Lipofectamine RNAi Max (Invitrogen, Thermo Fisher Scientific, Inc., Waltham, MA, USA). Briefly, siRNAs, diluting in the 100 μl Opti-MEM (Gibco, USA) at 5 nM and mixed with 5 μl Lipofectamine RNAi Max, were added to the cell culture medium, and the cells were collected after 48 h. The sequences of specific siRNAs are listed in Supplementary Table S1. Overexpression plasmids were constructed using pEZ-Lv201. Transfection of plasmids was performed using Lipofectamine 3000 (Invitrogen). Briefly, 2 μg plasmids and 5 μl p3000 were diluted in 150 μl Opti-MEM and 5 μl lipo3000 were diluted in 150 μl Opti-MEM. Then, the plasmids and lipo3000 solution were mixed and incubated at room temperature for 15 min. Finally, the mixture was added onto 6 well plates, and cells were collected after 48–72 h.

### 2.3 Animal studies model

BALB/c male nude mice (5–6 weeks old) were purchased from SPF (Beijing) Biotechnology Co., Ltd. (Beijing, China) and maintained in SPF facilities. The Institutional Ethical Board approved animal studies of the First Affiliated Hospital of Sun Yat-sen University.

For the popliteal lymph node metastasis model, MKN45 cells (Luciferase labeled, 1 × 10^6^ in 20 μl PBS) were injected into the left footpad of mice and eight nude mice per group were used in the experiment. Six weeks after injection, fluorescein sodium salt (Solarbio) was injected into the tail vein of the mice. The bioluminescence images were captured using an IVIS Lumina II (Xenogen Corp.). Then the left footpad and popliteal lymph nodes were resected, photographed, and fixed in 4 % paraformaldehyde for further analysis.

### 2.4 RNA extraction and qPCR analysis

According to the manufacturer's instructions, total RNA was extracted from cells and tissues using RNAiso (Takara, Kusatsu, Japan). Briefly, the cells in 6 well plates were lysed in 500 μl RNAiso on ice and then mixed with 200 μl chloroform(Baishi, Guangzhou, Guangdong, China). After incubating for 10 min on ice, the mixture was centrifuged at 12000 rpm for 15 min, and the top layer of RNA was collected. Next, 500 μl isopropanol (Baishi) was added to the mix and incubated for 10 min. After centrifugation at 12000 rpm for 10 min, the supernatant was discarded, and the pellet at the bottom of the tube was retained. Then, the RNA precipitate was washed with 75 % ethyl alcohol (Baishi) and diluted in DEPC water (Takara).

As instructed, quantifications of specific RNA were used SYBR® Green I (Accurate Biotechnology, Changsha, Hunan, China). Briefly, 5 μl SYBR Green, 3 μl DEPC water, 1 μl primers and 1 μl cDNA were prepared for each sample, and the qPCR protocol was run using a LightCycler® 480 II (Roche, Basel, Switzerland). The fold change was calculated using the relative quantification method (2^−ΔΔCt^). The qPCR primers used are listed in Supplementary Table S1.

### 2.5 Cell proliferation, migration, and invasion assays

For the cell proliferation assays, the cells were seeded in 96-well plates at 1000 cells per well. Then the CCK-8 (SolarBio, Beijing, China), diluted in standard culture media, was added into each well for 4 h. Proliferation rates were determined at 0, 24, 48, 72, and 96 h after seeding, and quantification was performed on a microtiter plate reader (ALLSHENG, Hangzhou, Zhejiang, China) measured at UV wavelength (λ) = 450 nm. For the cell migration and invasion transwell assays, 50 000 cells in 400 μl rich media were plated on the top chambers of Transwell Clear Polyester Membrane Inserts (for the migration assay, Corning Costar, New York, USA) and BioCoat Matrigel Invasion Chambers (for the invasion assay, Corning Costar), while culture media with 10 % FBS was applied to the bottom. After 16–72 h, migrated or invaded cells were stained with crystal violet and counted under a microscope (Olympus, Tokyo, Japan).

### 2.6 Immunohistochemistry (IHC)

Formalin-fixed, paraffin-embedded specimens were cut into 3-μm-thick sections and mounted onto adhesion microscope slides. After being deparaffinized and rehydrated, the 0.01 mol/L EDTA buffer (pH 8.0) was used for antigen retrieval. Sections were incubated with 3.0 % hydrogen peroxide (H_2_O_2_) solution to block endogenous peroxidase activity. Then the 5 % goat serum diluted in PBST (0.3 % Triton X-100 in PBS; Gibco) was used to block non-specific staining. After that, sections were incubated with diluted antibodies at 4 °C overnight. After rinsed and sequential 1-h incubations with horseradish peroxidase-conjugated secondary antibody (G1214; Servicebio, Wuhan, Hubei, China), targeted protein was visualized using liquid DAB (Servicebio).

### 2.7 Western blotting analysis

Western blotting analysis was performed as described previously. Briefly, equal amounts of protein were separated by 10 % or 12.5 % sodium dodecyl sulfate‐polyacrylamide gel electrophoresis (SDS‐PAGE) and transferred onto polyvinylidene difluoride (PVDF) membranes (Thermo Fisher Scientific, Inc.). Membranes were incubated with primary antibodies at 4 °C overnight and then secondary antibodies at room temperature for 2 h. After washing, signals were detected using a ChemiDoc™ imaging system (Bio‐Rad Laboratories, Inc.).

### 2.8 Cell death detection

The cells were cultured in a 6-well plate until they reached 60 %–70 % confluency. Before the experiments, drug treatment was performed as described before. The cells were then collected and centrifuged at 2000 rpm and washed twice with PBS. Finally, the cells were stained using the PI staining (Yishan, Shanghai, China) for 30 min and detected with CytoFLEX (Beckman Coulter, Brea, CA, USA).

### 2.9 BODIPY 581/591 staining

The cells were cultured in a 6-well plate until they reached 60 %–70 % confluency. Then the cells were treated as experiment requirements. The BODIPY 581/591 dye was diluted in completely culture medium at 1:1000 concentration and incubated with cells for 30 min. The cells were then collected and centrifuged at 2000 rpm and washed twice with PBS. BODIPY 581/591 staining was observed using BX63 microscope (Olympus).

### 2.10 Chromatin immunoprecipitation (ChIP)

The ChIP assay was performed using a ChIP assay kit (SimpleChIP® Plus Sonication Chromatin IP Kit #56383, Cell Signaling Technology, MA, USA). Briefly, GC cells were fixed with 1 % formaldehyde (Baishi) and quenched with glycine (#G8200, Solarbio) at room temperature, after which they were collected, washed, and resuspended in lysis buffer (Cell Signaling Technology). The sonicated chromatin solution was immunoprecipitated with a target antibody or a negative anti-IgG antibody. The immunoprecipitated DNA was purified by column collection and analyzed by RT-qPCR. The enrichment percentage was calculated using the relative quantification method 2 (CT^ChIP^ - CT^input^).

### 2.11 Untargeted sequence of metabolism

Novogene Co., Ltd. (Beijing, China) performed the untargeted metabolomic sequencing. Briefly, the samples were placed in EP tubes and resuspended in pre-chilled 80 % methanol (Baishi) and 0.1 % formic acid (Rhawn, Shanghai, China) by vortexing. The samples were melted on ice and washed for 30 s. After sonication for 6 min, the samples were centrifuged at 5000 rpm at 4 °C for 1 min. The supernatant was frozen, dried, and dissolved in 10 % methanol. Finally, the solution was injected into the LC-MS/MS system (SCIEX Headquarters, Framingham, MA, USA).

### 2.12 Single cell sequencing (sc-seq) analysis

Sc-seq data was downloaded from the SRA database (PRJNA776683) [33] and single-cell sequencing data were merged using Seurat. After removing the poor-quality cells and genes, gene expression was standardized and normalized. The dimensionality of the genes was reduced using principal component analysis (PCA), and the top 20 principal components were further analyzed using UMAP. Seurat used "Tobit" to calculate differential expression analysis, specifying that the percentage of cells in which a gene is detected in a particular cluster is >25 %. DEG was defined as |fold change| > 2, P < 0.05. Annotation clusters with the genetic markers EPCAM and CD24 were defined as tumor cells. Monocle 2 constructed single-cell pseudo-time trajectories. To identify the genes that separate cells into different states, we used the "BEAM" function. Genes with q values less than 0.01 obtained from BEAM analysis were separated using hierarchical clustering.

### 2.13 Statistical analysis

All statistical values were calculated using Statistical Product and Service Solutions (SPSS) 22.0 (Chicago, IL, USA). Experimental studies were analyzed by independent sample *t*-test to compare two groups and by one-way ANOVA followed by posthoc Tukey's test to compare multiple groups. Kaplan-Meier analysis and log-rank tests were used to evaluate differences in patient survival. The Spearman rank correlation test was used to determine statistical correlations. Statistical significance was set at *P* < 0.05.

## 3 Results

### 3.1 Draining lymph nodes of GC have a lipid-enriched microenvironment

**Fig. 1:**
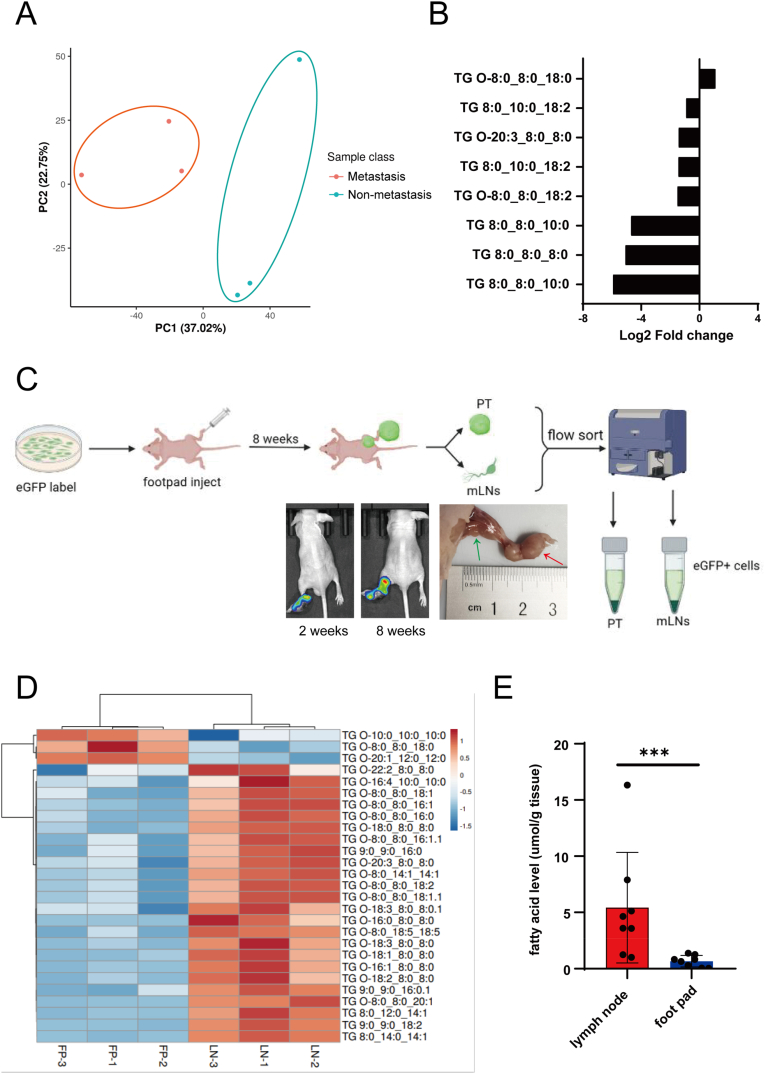
**Lymph nodes had lipid-enriched microenvironment.** A. PCA plot of lipid profile of GC tumor samples between N0 and N+ stages; B. The fold change of triacylglycerol in GC tumor samples between N0 and N+ stages; C. The diagram showed the flow of animal experiments; D. The heatmap of TG level in footpads and lymph nodes from mice models; E. ELISA analysis detected the total fatty acid level of footpads and lymph nodes. N0: lymph nodes metastasis negative; N+: lymph nodes metastasis positive; TG: triacylglycerol; FT: footpad; LN: lymph node; PT: primary tumor.

Lipids are among the most important metabolite families involved in energy supplementation and signal transduction. Therefore, we investigated the role of lipid metabolism in GC progression. GC tumors tissue and Adjacent normal mucous tissue were collected from three patients at the N0 stage and three patients at the N+ stage, respectively. We performed Non-target lipidomics sequencing on 12 samples obtained from 6 patients (including 6 tumor tissue samples and 6 adjacent normal mucous tissue tissue). The screening of different metabolites showed that the lipid profiles of tumors were distinct from those of normal tissues (Supplementary Fig. 1A and B). Furthermore, we explored whether the lipid profile of primary GC changed as the N stage advanced. Comparing lipid abundance between N0 and N+ stages across 6 tumor tissue samples, the principal component analysis (PCA) plot showed that the dots from the two groups were completely divided, indicating a shift in lipid metabolism when lymphatic metastasis occurred (Fig. 1A). In contrast, the levels of triacylglycerol (TG) were reduced in GC with LN metastasis (Fig. 1B). Collectively, these findings suggest that primary GC upregulates lipid consumption during LN metastasis.

Owing to the difficulty of gaining tumor cells from metastatic LNs from clinical patients, we employed animal models to investigate the reprogramming of lipid metabolism between tumors in the primary site and lymph nodes. EGFP-Luc-labeled MKN45 cells were injected into the right footpad of nude mice to establish a footpad-popliteal lymph node metastasis model. Eight weeks after injection, bioluminescence images showed that GC cells had colonized the popliteal lymph node. The mice were sacrificed, and the primary tumor and homolateral popliteal lymph nodes were harvested for further analysis (Fig. 1C). Non-targeted lipidomic sequencing was performed to explore the discrepancies in the lipid microenvironment. There were 324 downregulated and 186 upregulated metabolites in primary footpad tumors compared to metastatic LNs, including fatty acids, sphingolipids, and glycerophospholipids (Supplementary Fig. 1C). The heatmap shows a prominent difference in lipid metabolites between the two tissues (Supplementary Fig. 1D). Among the changing metabolites, triglycerides, the primary lipid storage form, were significantly increased in the lymph nodes (Fig. 1D). The fatty acid, which are critical substrates for beta-oxidation, lipid biosynthesis, and transport, represent the form directly utilized by cells for these metabolic processes. However, the lipidomic sequencing annotations did not include fatty acids. To address this limitation, we measured total fatty acid levels using an ELISA kit. The findings revealed a significant increase in total fatty acid levels in the lymph nodes compared to that at the primary site (Fig. 1E). These results suggest that lymph nodes have a high lipid-rich microenvironment compared to the primary site.

### 3.2 Lipid metabolism of GC is inhibited after lymph node metastasis

**Fig. 2:**
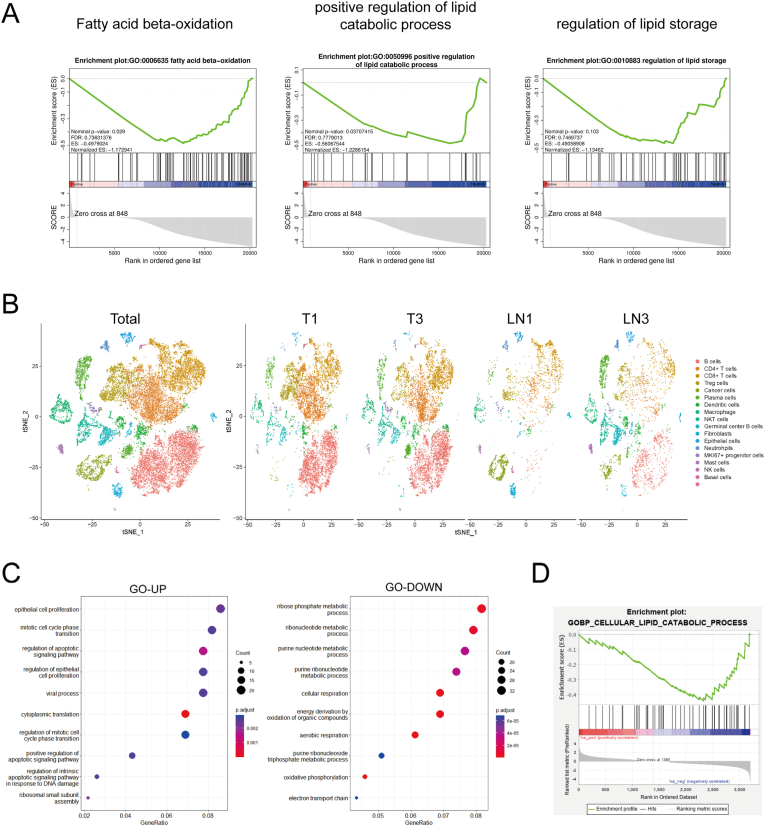
**Lipid metabolism of GC was inhibited after LN metastasis.** A. GSEA analysis of different expression genes (DEG) between GC cells from PT and LN; B. Uniform manifold approximation and projection (UMAP) plot showing graph-based clustering of isolated cells from PT and LN colored by clusters and their annotations; C. GO analysis of DEG from GC cells in sc-seq data; D. GSEA analysis of lipid catabolic pathway.

To explore the reprogramming of lipid metabolism in GC cells after metastasis to the lymph nodes, we separated GC cells using an eGFP label. Fluorescence microscopy of frozen sections of primary footpad tumors and metastatic LNs showed that GC cells expressed certain eGFP signals in both tissues (Supplementary Fig. 2A). After digesting the fresh tissues into a single-cell solution, flow cytometry was used to isolate eGFP-positive cells to separate the GC cells (Fig. 1C). RNA sequencing was performed to identify differentially expressed genes (DEGs) in gastric cancer cells from mouse footpad tumors and metastatic LNs. There were 586 upregulated and 992 downregulated genes in GC cells in the LN compared to those in the footpad (Supplementary Fig. 2A). GO analysis showed that DEGs were significantly enriched in metabolic pathways (Supplementary Fig. 2C). Subsequently, GSEA revealed that fatty acid beta-oxidation, lipid catabolic processes, and lipid storage pathways were suppressed in LNs (Fig. 2A). Collectively, the footpad-popliteal metastasis model established inhibition of lipid catabolism and storage of GC in the LN microenvironment.

Moreover, we used sc-seq RNA data of patients with GC from the SRA database (PRJNA776683) to explore whether suppression of lipid metabolism was present in clinical samples. We obtained the transcriptional profiles of 23 888 cells after quality control and separated the cells into 20 clusters using PCA and UMAP analysis (Fig. 2B). Guided by the top marker genes, we identified clusters of known cell types, including B cells (CD19), granulocytes (S100A8), monocytes (MS4A7), regulatory T cells (CD25), cytotoxic T cells (CD8), lymphatic endothelial cells (VEGFR2), plasma cells (DERL3), and dendritic cells (FCER1A and CST3). To further analyze the lipid metabolism pathway in GC cells, we identified tumor cells using the GC-specific markers EPCAM and CD24 (Fig. 2B). Differentially expressed genes (DEGs) were compared between GC cells at the primary site and LNs. GO analysis revealed that upregulated genes were mostly associated with cell proliferation and cell death pathways, whereas downregulated genes were associated with purine metabolism and cell respiration (Fig. 2C). GSEA also confirmed that the lipid catabolic pathway was inhibited in LN-derived GC cells (Fig. 2D). Most importantly, both the footpad-popliteal metastasis model and sc-seq suggested that GC cells suppress lipid metabolism when they colonize LNs.

### 3.3 Arachidonic acid increases ferroptosis sensitivity of GC

Interestingly, GC cells were dispersed in a relatively high-lipid microenvironment while suppressing the lipid metabolism pathway. This finding seems to contradict those of previous studies, which reported that lipid oxidation fuels cancer metastasis [[14], [15], [16]]. Based on this observation, we hypothesized that GC cells suppress lipid metabolism to mitigate cell death induced by high lipid levels in the LN microenvironment.

**Fig. 3:**
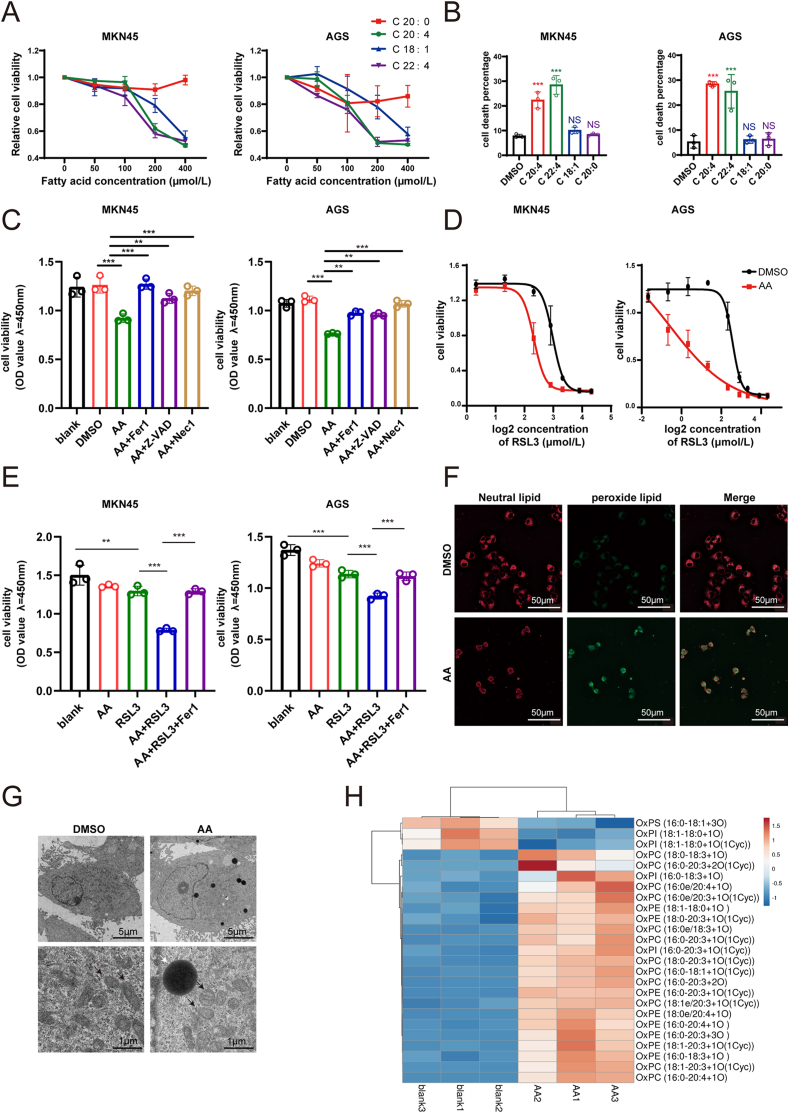
**Arachidonic acid (AA) increased ferroptosis sensitivity of GC.** A. CCK-8 analysis detected the cell viability under fatty acid treatment; B. Flow cytometry tested cell death percentage of GC cells treated with fatty acid at 100 μmol/L concentration; C. CCK-8 analysis detected the changing of cell viability after using cell death inhibitors,AA:100μM, Fer1:10μM, Z-VAD:5uM, Nec1:1uM; D. Drug susceptibility curve of Rsl-3 with or without AA treatment; E. CCK-8 analysis detected the cell viability under AA(50μM), Rsl3(2.5-5μM) and Fer1(10μM) treatment; F. Fluorescence images of BODIPY 581/591 staining, scale bar: 50 μm; G. Electron microscope images of lipid droplets and mitochondria in GC cells, White arrows indicate lipid droplets and black arrows point to mitochondria, scale bar: 5 μm (upon) and 1 μm (bottom); H. The heatmap of oxidized lipid in GC cells measured by lipidomic sequencing.

Fatty acids, including saturated and mono/polyunsaturated fatty acids, are directly utilized by cells from the microenvironment for beta-oxidation or lipid biosynthesis [34]. In this study, we detected the cytotoxicity of four fatty acids (arachidic acid, C 20:0; oleic acid, C 18:1; arachidonic acid, C 20:4; and docosatetraenoic acid, C22:4; Supplementary Fig. 3A). The dose-inhibition curves showed that saturated fatty acid exhibited minimal cytotoxic, while oleic acid showed modest cytotoxic and polyunsaturated fatty acid-induced significant cell death at the concentration of 200–400 μmol/L in MKN45, AGS and HGC27 cells (Fig. 3A, Supplementary Fig. 3B). Flow cytometry analysis further confirmed that only polyunsaturated fatty acids induced dramatic cell death at 100 μmol/L concentration (Fig. 3B, Supplementary Fig. 3C and D). Notably, high concentrations of polyunsaturated fatty acids induce cell death. However, CCK-8 assay showed that ferroptosis inhibitor Fer-1, apoptosis inhibitor Z-VAD and the necrosis inhibitor Nec-1 partially or completely restored cell viability treatment with 100 μmol/L AA (Fig. 3C, Supplementary Fig. 4A); this findings indicate that high concentration of AA induces GC cell death in a nonprogrammed manner.

While direct measurement of physiological AA concentrations in tumor microenvironments through lymph fluid mass spectrometry would be ideal, this procedure requires specialized clinical sampling that was not feasible within our current study framework. To address this important point, we have now performed comparative AA level analysis using ELISA assays on paired specimens from 3 gastric cancer patients (primary tumors vs metastatic lymph nodes (mLN)). The data demonstrate a statistically significant increase of AA levels in metastatic lymph nodes compared to primary lesions (Supplementary Fig. 4B). However, achieving an AA concentration exceeding 100 μmol/L in physiological microenvironment is nearly impossible [17]. Thus, we investigated the cytotoxic effect of AA at a concentration of 50 μmol/L as reported in previous studies. The CCK-8 results showed that AA treatment at 50 μmol/L significantly increased the sensitivity of GC cells to the ferroptosis inducer RSL3 (Fig. 3D, Supplementary Fig. 4C). In addition, PI staining and CCK-8 results confirmed that while 50 μmol/L AA alone did not induce cell death, it increased cell death upon RSL3 treatment. The ferroptosis-promoting effect of AA was partially reversed by FER-1 treatment (Fig. 3E, Supplementary Fig. 4D and E). Furthermore, The RNA-seq was performed on MKN45 cells treated with AA. GO analysis confirmed that the DEGs were enriched in the ferroptosis and AA metabolism pathways (Supplementary Fig. 4F). Additionally, GSEA analysis revealed that AA promotes ferroptosis and suppresses lipid biosynthesis (Supplementary Fig. 4G).

We examined the ferroptotic phenotype of GC cells following AA treatment. BODIPY 581/591C11 staining revealed that peroxide lipids were enriched in the GC cells following AA treatment (Fig. 3F, Supplementary Fig. 5A). Transmission electron microscopy revealed the presence of lipid droplets and swollen mitochondria with fractured cristae in GC cells following AA treatment (Fig. 3G, Supplementary Fig. 5B). Lipidomic sequencing also showed that AA treatment increased the total and oxidized lipid levels in GC cells (Fig. 3H). Collectively, these findings suggest that while the physiological concentration of AA did not directly induce cell death, it enhanced the ferroptosis sensitivity of GC.

### 3.4 FABP1 was downregulated in LN metastatic GC cells

**Fig. 4:**
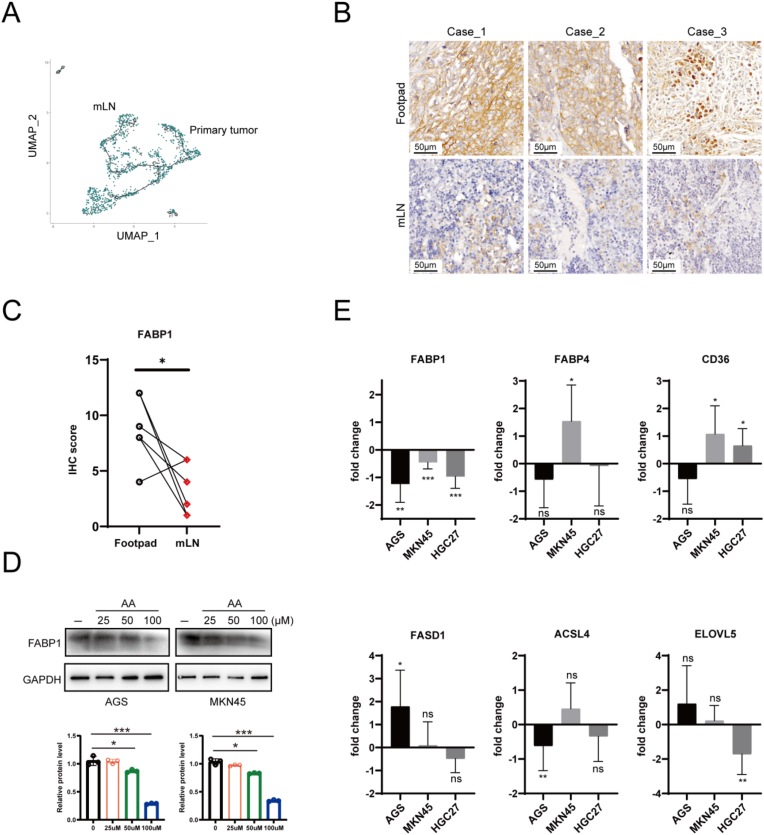
**FABP1 was downregulated in LNs.** A. The single-cell trajectory plots by Monocle 3. The trajectory contains two main branches; B. IHC staining FABP1 of footpad and LNs, scale bar: 50 μm; C. IHC score of each image in Fig. 4B; D. Western blotting results and band density analysis showed the FABP1 expression under AA treatment; E. RT-qPCR detected the fold change of six genes expression under AA treatment.

We further investigated the suppression of lipid metabolism in GC in the LNs using sc-seq data (Fig. 2C). Monocle 3 analysis was applied to construct a trajectory of the identified GC cells using the entire set of genes to address the transformation process of GC cells from PT to mLN. The trajectory mainly contained two branches and the cells were grouped into three states (Fig. 4A). In the Monocle 3 analysis, genes with similar patterns of expression that varied over time across the pseudotime trajectory were coalesced into modules (Supplementary Fig. 5C). Among these, the fatty acid transport protein FABP1 was categorized in module 47, which was downregulated in LNs compared to the primary site (Supplementary data 1). In this study, we examined the expression levels of FABP1. IHC staining of tissues from the footpad-popliteal metastasis model corroborated these findings, showing higher FABP1 expression in footpad tissues than in lymph nodes. (Fig. 4B and C). These findings indicate that FABP1 expression is inhibited in LN metastatic GC cells.

Given the high concentration of AA in LN, we investigated whether microenvironmental fatty acid suppresses FABP1 expression in GC cells. Western blot analysis revealed that AA treatment dramatically impaired FABP1 expression in GC cells (Fig. 4D). Moreover, AA treatment specifically inhibited FABP1 expression without affecting the expression of other lipid metabolism-related genes, such as CD36, FABP4, and ACSL4, which are involved in ferroptosis (Fig. 4E). These findings suggest that the LN microenvironment contributes to the suppression of FABP1 expression in GC cells.

### 3.5 FABP1 mediates AA uptake promotes ferroptosis sensitivity of GC cells

Our findings revealed that FABP1 expression was negatively correlated with LN metastasis in GC. However, evidence supporting the functional role of FABP1 in GC cells remained limited. Combining the RNA profiles of GC and normal tissues from the TCGA and GTEx databases, we found that FABP1 was highly expressed in tumors tissues (Supplementary Fig. 6A). However, FABP1 overexpression in AGS and MKN45 cells did not promote cell proliferation or migration (Supplementary Fig. 6B and C). In contrast, GC patients with high FABP1 expression showed better long-term outcomes (Supplementary Fig. 6D), supporting the association between FABP1 and cell death. Correlation analysis of RNA levels from the TCGA database also confirmed the negative correlation of FABP1 and GPX4, a negative regulator of ferroptosis, in GC (Supplementary Fig. 6E). Thus, we hypothesized that FABP1 plays a critical role in the ferroptosis of GC cells.

**Fig. 5:**
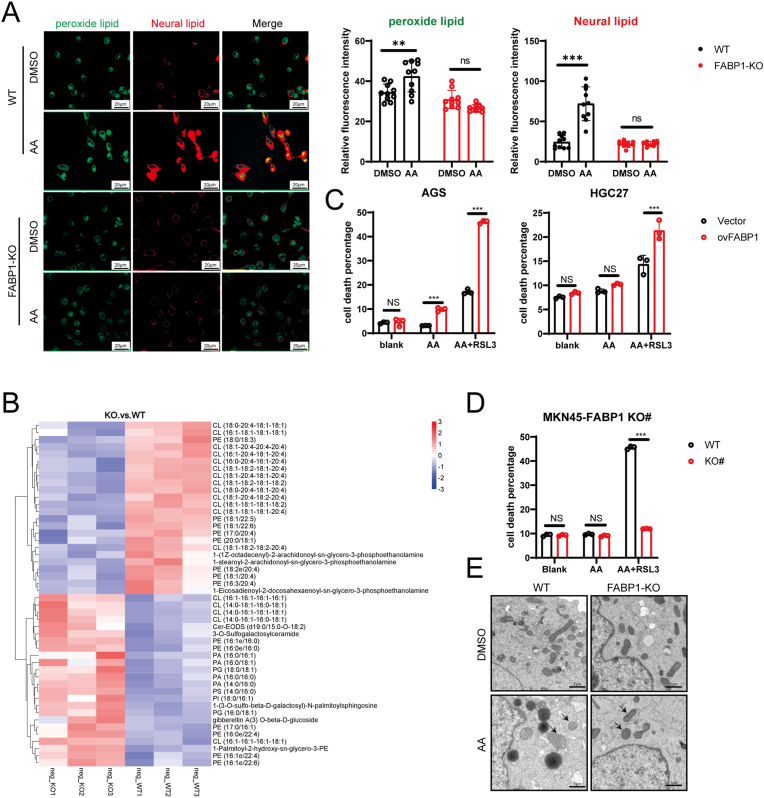
**FABP1 mediated AA uptake and ferroptosis sensitivity.** A. BODIPY 581/591 staining identified the peroxide lipid (Green) and neural lipid (Red) in GC cells; B. Heatmap of the enrichment changed lipid in GC cells on FABP1 knockout; C-D. Flow cytometry detected cell death percentage in FABP1 overexpression cells (C) and knockout cells (D),AA:50μM, RSL3:1-5μM; E. electronic microscope images of lipid droplets and mitochondria, White arrows indicate lipid droplets and black arrows point to mitochondria, scale bar: 1 μm.

To investigate the role of FABP1 in ferroptosis, we used the CRSPR-Cas9 system to knock out FABP1 in MKN45 cells. After drug screening and clone formation, clone #5 had the best efficiency of FABP1 knockout, as confirmed by western blotting (Supplementary Fig. 6F). Sanger sequencing verified that ddATP was deleted in the sgRNA fragment (Supplementary Fig. 6G). Previous studies have reported that AA treatment increases lipid peroxidation in GC cells [35]. As expected, knockdown FABP1 inhibited lipid enrichment and peroxidation in AA-treated MKN45 cells (Fig. 5A). Furthermore, the level of AA contained lipids was lower in FABP1 knockout cells compared to in wild-type (WT) cells with AA treatment, as detected by lipidomic sequencing (Fig. 5B). The ELISA results also demonstrated that FABP1 knockdown reduced the relative levels of arachidonic acid (AA) in MKN45 cells (Supplementary Fig. 6H). These data indicate that FABP1 mediates AA uptake by GC cells.

Moreover, the overexpression of FABP1 in AGS and HGC27 cells significantly increased the cell death percentage with AA + RSL3 treatment while having no effect in the blank control or AA treatment groups (Fig. 5C, Supplementary Fig. 6I). In contrast, FABP1 knockout had the opposite effect (Fig. 5D). ATP assay revealed that under AA treatment, the FABP1 knockdown group exhibited higher ATP levels. Flow cytometry analysis of JC-1 further demonstrated that the FABP1 knockdown group had a lower proportion of dead cells, suggesting that FABP1 deficiency mitigates AA-induced mitochondrial dysfunction (Supplementary Fig. 6J and K). Transmission electron microscopy revealed that FABP1 knockout rescued lipid droplet accumulation and structural destruction of mitochondria in AA (Fig. 5E). In summary, FABP1 mediates AA uptake and controls the ferroptosis sensitivity of GC cells under AA conditions.

### 3.6 Downregulated PPARG pathway inhibited FABP1 transcription of GC in LNs

**Fig. 6:**
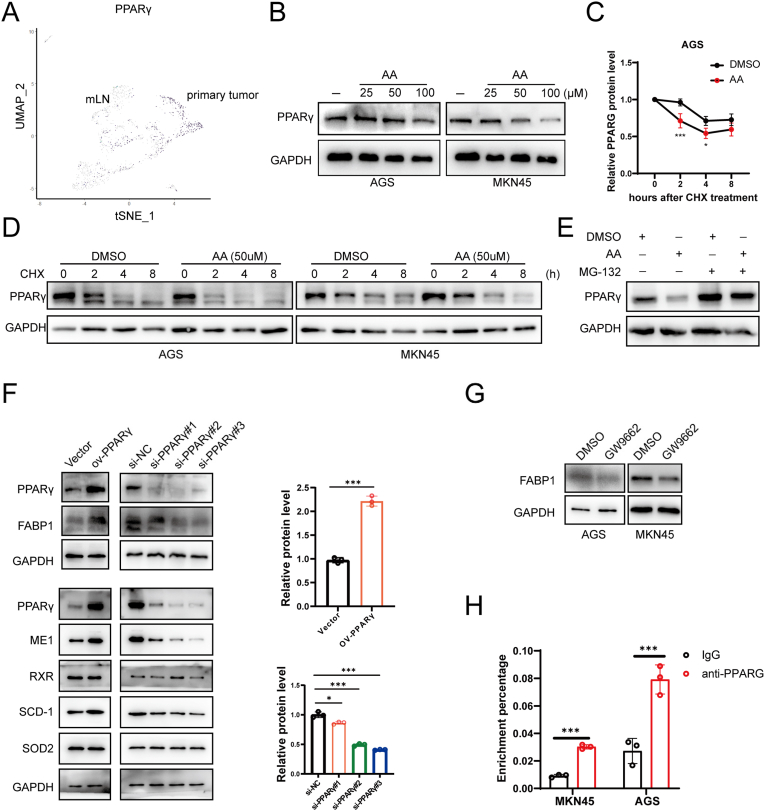
**Suppression PPARG decreased FABP1 transcription in GC.** A. PPARG expression in each cells on single-cell trajectory plots; B. PPARG expression under AA treatment; C. Gray value analysis of PPARG degradation on AA treatment, AA:50μM; D. Western blotting images of PPARG degradation on AA treatment; E. Western blotting detected PPARG level on AA and MG-132 treatment; F. Western blotting results and band density analysis showed FABP1 protein levels in GC cells with PPARG overexpression and knockdown; G. FABP1 levels on GW9662 treatment; H. ChIP-qPCR analysis identified the enrichment of FABP1 sequencing of PPARG antibody.

Since FABP1 was downregulated in LN metastatic GC cells, we explored the mechanism of FABP1 inhibition by reanalyzing the sc-seq data presented in Fig. 2. DEG analysis revealed that the lipid metabolism regulator PPARG was significantly downregulated in LN-metastatic GC cells (Fig. 6A, Supplementary Fig. 7A). GSEA analysis confirmed inhibition of the PPARG pathway in GC after LN metastasis (Supplementary Fig. 7B). Since AA treatment can inhibit the expression of FABP1 protein, we next investigated whether AA directly suppressed the expression of PPARG protein in GC cells. Previous studies have reported that AA derivatives are PPARs agonists in eukaryotic cells; however, no evidence has shown that AA inhibits PPARG expression. After treating GC cells with progressive AA concentration, the western blotting showed PPARG protein level was downregulated in over 50 μM AA condition (Fig. 6B). Additionally, AA treatment decreased PPARG protein stability in GC cells, as detected by the CHX assay (Fig. 6C and D, and Supplementary Fig. 7C). The proteasome inhibitor MG-132 abolished the suppressive effect of AA on PPARG protein expression (Fig. 6E). Thus, it appears that extremely high AA concentrations can downregulate PPARG expression.

Subsequently, we investigated whether the PPARG pathway directly modulated FABP1 expression in GC cells, and we also verified changes in PPARG's known downstream molecules SCD-1, ME1, and its partner protein RXR. The results showed that Overexpression of PPARG significantly promoted the expression of FABP1, SCD-1, and ME1 in GC cells, while RXR protein expression remained unaffected. In contrast, the knockdown of PPARG with specific siRNAs dramatically inhibited the protein expression of FABP1, SCD-1, and ME1, with similarly negligible changes observed in RXR. Previous studies have reported that PPARG interacts with FABP1, and this interaction is enhanced during inflammation, while SOD2 deficiency can induce AA production and promote inflammation [29,36]. However, our results demonstrated that SOD2 expression was not affected by PPARG protein. (Fig. 6F). Similarly, PPARG pathway inhibitor GW9662 suppressed the RNA levels of FABP1 and other well-known PPARG receptor genes in GC cells (Supplementary Fig. 7D). Western blotting further confirmed that GW9662 effectively inhibited FABP1 expression in GC cells (Fig. 6G). However, the results of the flow cytometry analysis show that GW9662 cannot save AA-induced ferroptosis (Supplementary Fig. 7E). Because PPARG functions as a transcription factor in eukaryotic cells, the JASPR online tools were used to predict the direct interaction sites between PPARG and the FABP1 regulatory region (Supplementary Fig. 7F). The results identified three binding sites in the sequence located 1000 bp upstream of the FABP1 gene. To validate these findings, we performed ChIP assay to detect the combined activity of the PPARG and FABP1 promoter regions. As shown in (Fig. 6H and Supplementary Fig. 7G), the PPARG antibody significantly enriched the DNA fraction of the FABP1 regulatory region compared to negative IgG. Thus, the PPARG pathway directly promotes FABP1 transcription in GC cells.

### 3.7 Knocking out FABP1 increased lymph node metastasis of GC in vivo

**Fig. 7:**
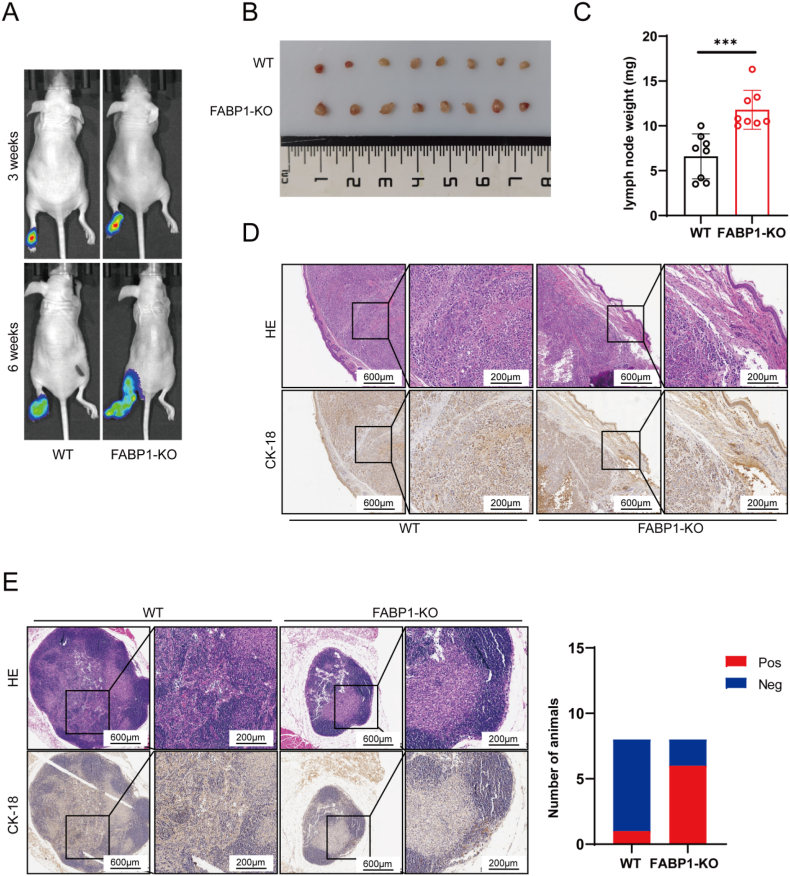
**FABP1 knockout improved LN metastasis of GC.** A. luminescence images showed the GC in footpad and popliteal LNs in mice; B. Harvested popliteal LNs from mice after 6 weeks injection; C. The LNs weight of harvested LNs; D. H&E and IHC of CK-18 images of footpad tumor, scale bar: 600 μm (left), 200 μm (right); E. H&E and IHC of CK-18 images of popliteal LNs (left) and number of animals with CK-18 positive or negative staining in metastatic lymph nodes per experimental group (right).

To determine whether FABP1 attenuates LN metastasis in vivo, we constructed a footpad lymph node metastasis model using FABP1-KO MKN45 cells and the corresponding control. Six weeks after injection, FABP1-KO cells had more serious migration into the ipsilateral popliteal LNs than the control, as reflected by bioluminescence imaging (Fig. 7A). Harvested LNs from mice injected with FABP1-KO cells had larger tumor volumes and higher tumor weights (Fig. 7B and C). To detect tumor lesions in the LNs, we performed H&E and IHC staining of CK-18 in both footpad and LNs tissues. Consistent with previous reports [37], our IHC images of footpad tissues further confirmed CK-18 as a reliable biomarker for GC cells (Fig. 7D). IHC and H&E images of LNs showed that FABP1-KO significantly reduced the metastatic lesion area and frequency compared to the control (Fig. 7E). FABP1 expression in GC cells inhibits tumor metastasis to LNs in vivo.

## 4 Discussion

Lymph node metastasis is an independent risk factor for GC long survival. Most patients are first diagnosed with secondary tumors in the local lymph nodes [38,39]. Unfortunately, there are limited treatment regimens targeting LN tumors. Lipids are among the most important energy resources for cellular activity and membrane structure [40]. Previous studies reported that cells exhibit elevated lipid metabolism during epithelial cells tumorigenesis [[41], [42], [43]]. In this study, our findings revealed that the lipid profile was significantly different between GC and the adjunct normal mucosa. The stored form of lipid TG decreases as the N stage of GC progresses. However, discovering the lipid discrepancy at the primary site is not sufficient to identify translatable metabolic vulnerabilities for further therapy. The influence of the microenvironment on metabolic features is important for exploring the mechanism of LN metastasis and potential therapeutic targets. Therefore, we constructed a footpad-popliteal metastasis model to imitate the microenvironmental shift when LN metastasis occurs. We further confirmed the lipid-enriched microenvironment in LNs compared with the primary site in vivo.

Compared to in situ tumor cells, metastatic cells require more energy supplements for ameboid motion and vascular invasion. Tumor cells overexpressing lipid use genes show improved migration and invasion abilities both in vitro and in vivo [15,[44], [45], [46]]. Distal tumor metastasis is a complex process in which tumor cells face various conditions. To explore whether GC cells maintain a high lipid metabolism pathway after colonization of lymph nodes, we identified the lipid metabolism features of LN tumor cells from both animal models and patients with GC. Both sets of sequencing data revealed that the lipid catabolism pathway was suppressed in LN tumor cells compared to that in primary situ. This finding, when considered alongside previous reports, raised a paradox: while tumor-induced lipid metabolism supports LN metastasis, it appears to suppress lipid catabiosis following colonization in a lipid-enriched microenvironment. To address this paradox, we investigated the effect of fatty acids on GC cells. CCK-8 and flow cytometry analyses suggested that high concentrations of PUFAs induce cell death in GC cells. However, it is difficult to determine the concentration at which PUFAs directly induce cell death. Moreover, PUFA, especially AA, increased ferroptosis sensitivity at 50uM concentrations. As is well known, ferroptosis is a form of cell death characterized by PUFA p eroxidation, that occurs under condition of cellular stress [47,48]. Ferroptosis plays a key role in regulating chemotherapy resistance, metastasis, and proliferation in GC cells [[49], [50], [51]]. Xin et al. reported that inhibiting ferroptosis in tumor cells improved lymph node metastasis in GC [52]. Based on these findings, we hypothesized that AA-induced ferroptotic stress in GC cells may explain suppression of the lipid metabolism pathway in LN microenvironment.

Furthermore, we identified FABP1 as a key protein that mediates AA uptake in GC cells. Sc-seq and IHC staining confirmed the downregulation of FABP1 in LNs. FABP1 is a fatty acid-binding protein involved in fatty acid uptake and transportation [53]. Given that disruption of FABP1 impairs fatty acid uptake by cells [30,54], FABP1 downregulation may reduce ferroptosis in GC cells. However, Ni ZH et al. reported that the downregulation of PPARα-FABP1 pathway induced cell ferroptosis which, contributed to immunoglobulin A nephropathy [55]. To investigate the relationship between FABP1 and ferroptosis in GC cells, we established FABP1 overexpression and knockout. As expected, FABP1 knockout suppressed AA-induced ferroptosis, whereas FABP1 overexpression had an inverse effect. Moreover, our findings revealed that FABP1 knockdown impaired AA uptake and peroxide lipid accumulation in GC cells. Collectively, GC cells showed decreased FABP1 expression to resist ferroptosis caused by AA in the LN microenvironment.

In addition, we observed that the PPARG pathway was downregulated in LN-derived GC cells. The PPARG pathway regulates several metabolic pathways, including lipid uptake, catabolism, and synthesis in human cells [56]. In this study, we found that PPARG acts as a transcription factor that directly induces FABP1 expression. Activation of the PPARG pathway is regulated by complex internal and external factors, and the AA metabolic derivative is an affinity antigen-activated PPARG protein [57]. However, the effects of fatty acids on PPARG pathway activation remain controversial. Palmitic acid could inhibit PPARG expression in a dose-dependent manner in hepatocyte cells [58]. In this study, we demonstrated that AA suppresses PPARG expression in GC cells in a dose-dependent manner. Collectively, we found that high concentrations of AA in LNs suppressed the PPARG pathway and inhibited FABP1 expression in GC cells. However, our result showed that inhibition of the PPARG pathway with GW9662 did not rescue the ferroptosis sensitivity induced by AA treatment. Previous studies have reported that PPARG pathway promotes ferroptosis resistance in various cancers [59,60]. We hypothesize that PPARG inhibition influences not only FABP1 expressions but also cell metabolic homeostasis due to the complex regulation of lipid metabolism and other interconnected pathways. Therefore, though GC cells suppress the PPARG pathway to inhibit FABP1 expression in LNs, inhibiting the PPARG pathway in vitro did not reduce AA-induced ferroptosis sensitivity. Fatty acid-binding proteins (FABPs, including FABP1-9 and FABP12) are members of a superfamily of intracellular lipid-binding proteins present in a variety of tissues. They play important roles in the uptake, transport and metabolic regulation of long-chain fatty acids. Currently, several novel drugs targeting FABPs have been developed for the treatment of obesity, atherosclerosis, diabetes, and cancer [61]. FABP5 inhibitors, such as the chemical inhibitors SBFI-26, SBFI-102, and SBFI-103, can inhibit malignant progression of prostate cancer [62]. Additionally, BMS309403, an inhibitor of FABP4, can increase the sensitivity of ovarian cancer to carboplatin [13]. Orlistat, a FABP1 inhibitor, limits tumor growth in hepatocellular carcinoma while enhancing anti-PD-1 function [29]. Although current therapeutic strategies targeting the FABP family primarily involve suppressing their expression—seemingly contradictory to our observation of FABP1 downregulation in metastatic lymph nodes of gastric cancer—similar paradoxical findings have been reported for FABP1 in colorectal cancer [63,64]. Notably, studies have demonstrated that loss of FABP1 in colorectal cancer correlates with increased lymph node metastasis [65]. Furthermore, reduced FABP1 expression has been documented in MSI-high (microsatellite instability-high) colorectal cancers, potentially attributable to the IFNγ-mediated suppression of PPARG within their highly immunoreactive microenvironment [66]. These collective findings suggest that FABP1-targeted cancer therapies may require context-dependent, multimodal intervention strategies. However, the effectiveness of these drugs in cancer treatment still needs further validation [67]. Therefore, we look forward to additional in vivo experiments with FABP1, which will ultimately benefit gastric cancer patients.

In conclusion, our study showed that a high concentration of AA can increase the sensitivity of gastric cancer cells to ferroptosis. Additionally, this study demonstrated that the lipid metabolism of gastric cancer cells was inhibited to adapt to the high-lipid microenvironment post-metastasis to lipid-rich lymph nodes. The lymph node microenvironment with high AA inhibited the PPARG-FABP1 pathway in gastric cancer cells, leading to reduced AA uptake and further contributing to ferroptosis resistance. The findings of this study identify potential therapeutic targets for LN metastatic GC and highlight the broader relevance of lipid-driven ferroptosis-resistance mechanisms in cancer biology.

## CRediT authorship contribution statement

**Yinan Liu:** Data curation, Investigation, Methodology, Writing – original draft, Writing – review & editing. **Liang Tang:** Data curation, Investigation, Methodology, Writing – original draft. **Baifu Peng:** Data curation, Investigation, Methodology, Writing – original draft. **Shaoji Zhao:** Investigation. **Ziling Shao:** Investigation. **Kaiyu Sun:** Investigation. **Jinning Ye:** Investigation. **Wei Chen:** Conceptualization, Project administration, Supervision, Writing – original draft, Writing – review & editing. **Jianbo Xu:** Conceptualization, Funding acquisition, Supervision, Visualization, Writing – original draft, Writing – review & editing.

## Data availability statement

The data that support the findings of this study are available from the corresponding author upon reasonable request.

## Funding

This work was supported by the 10.13039/501100001809National Natural Science Foundation of China (No.82273222, No.82473190, and No.82203642). the 10.13039/501100003453Natural Science Foundation of Guangdong Province, China (2022A1515012140).

## Declaration of competing interest

The authors declare that they have no known competing financial interests or personal relationships that could have appeared to influence the work reported in this paper.
