# Supplementary material for: Gastric cancer adapts high lipid microenvironment via suppressing PPARG-FABP1 axis after arriving in the lymph node
Source: Redox Biol. 2025 Jul 17;85:103759. doi: 10.1016/j.redox.2025.103759 (PMC12304710; doi:10.1016/j.redox.2025.103759)
Supplement: Multimedia component 1 [file mmc1.docx]

**Supplementary figures**


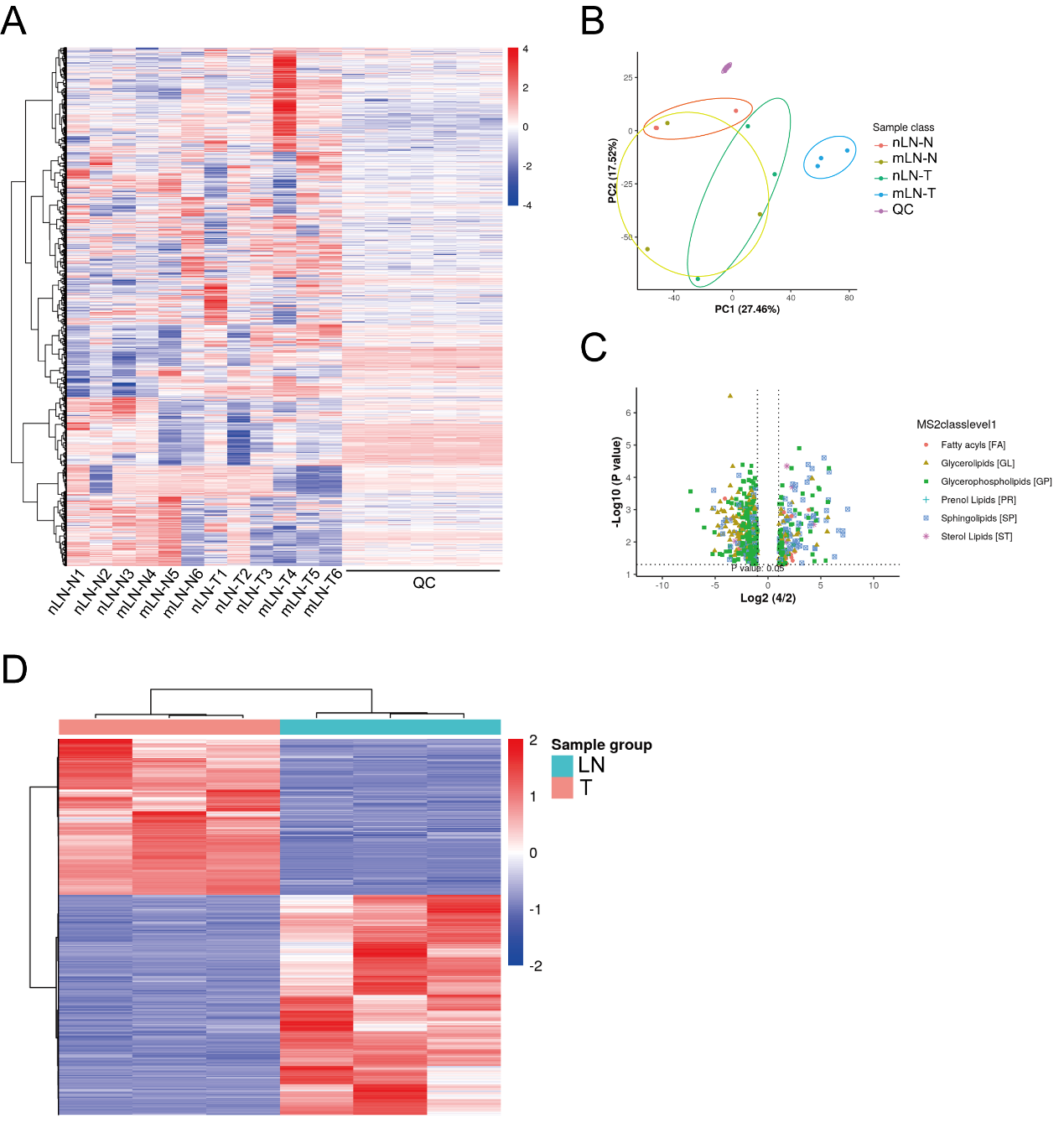


**Supplementary Figure 1. Non-targeted lipidomic sequencing screened different enriched lipids.** A. The heatmap of all mapped lipids in GC patient samples; B. PCA plot of lipid profiles in GC samples; C. Volcano plots showed different abundances of lipids between tumor tissues from footpad and lymph nodes in mice; D. Heatmap of lipids abundance difference between tumors from footpad and lymph nodes.

nLN: lymph nodes metastasis negative; mLN: lymph nodes metastasis positive; N: Adjacent normal tissue; T: tumor


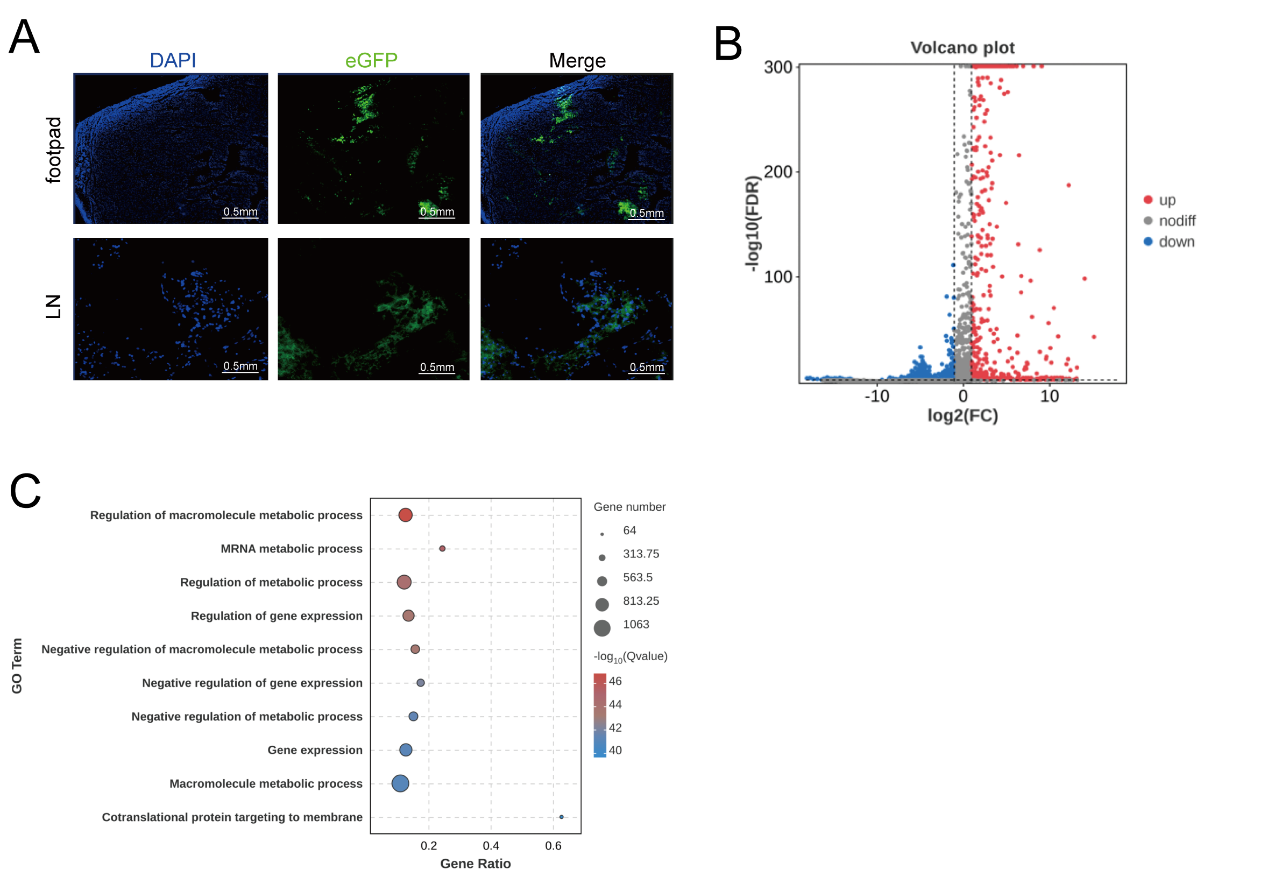


**Supplementary Figure 2. Different expression genes of GC cells in footpad and lymph nodes.**

A. Fluorescence images of frozen section from footpads and drain lymph nodes in mice, Green: eGFP positive GC cells; B. Volcano plot showed DEGs in GC cells; C. GO analysis of different expression genes in GC cells.

DEG: different expression genes


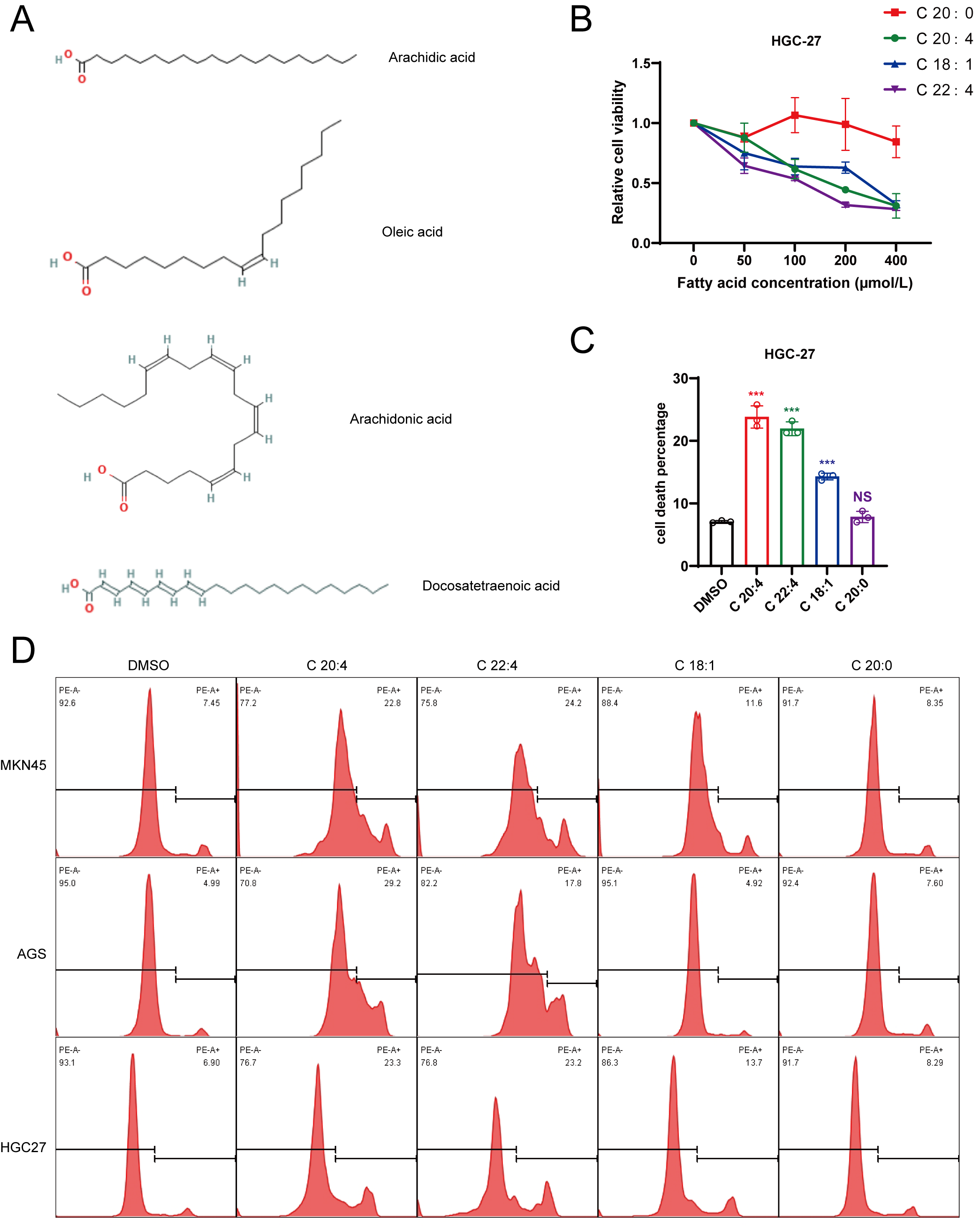


**Supplementary Figure 3. Fatty acid induced GC cell death.** A. Chemical structure formula of four kinds of fatty acids; B. CCK-8 analysis detected the cell viability of HGC27 under fatty acid treatment; C. Flow cytometry analysis showed cell death percentage of HGC27 under fatty acid treatment; D. Histogram of PI signal of GC cells under fatty acid treatment.

AA: Arachidonic acid


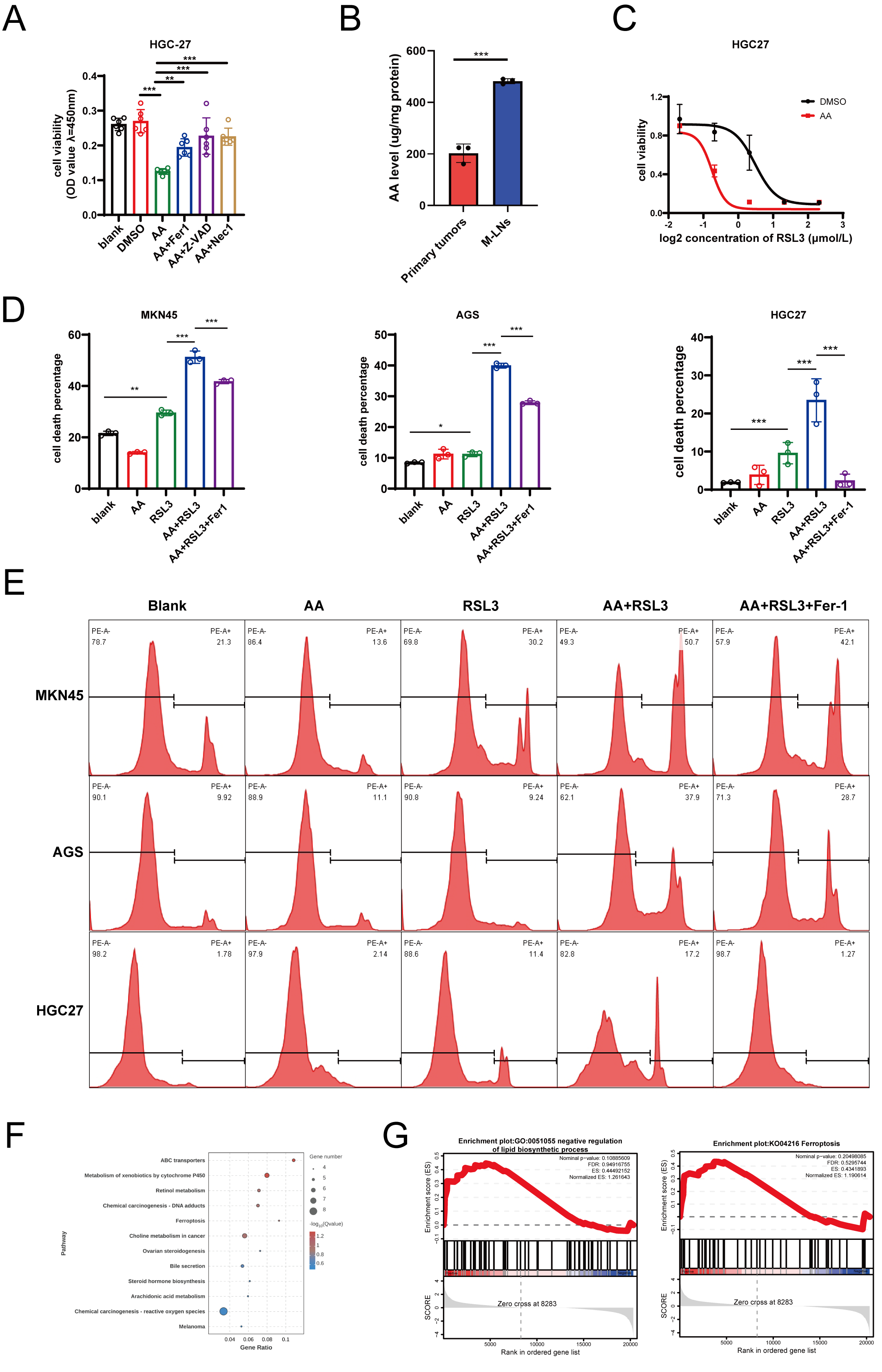


**Supplementary Figure 4. AA increased ferroptosis sensitivity of GC cells.** A. CCK-8 analysis detected cell viability of HGC27 under AA and cell death inhibitor treatment; B. ELISA analysis detected the Arachidonic acid level of Primary tumors and M-LNs. M-LNs: Metastatic lymph nodes; C. Drug susceptibility curve of Rsl-3 with or without AA treatment in HGC27; D. Flow cytometry detected the cell death percentage under AA, Rsl-3 and Fer-1 treatment; E. Histogram of PI signal of GC cells under AA, Rsl-3 and Fer-1 treatment; F. GO analysis of DEG between MKN45 cells with or without AA treatment; G. GSEA analysis of DEG in AA treated GC cells.


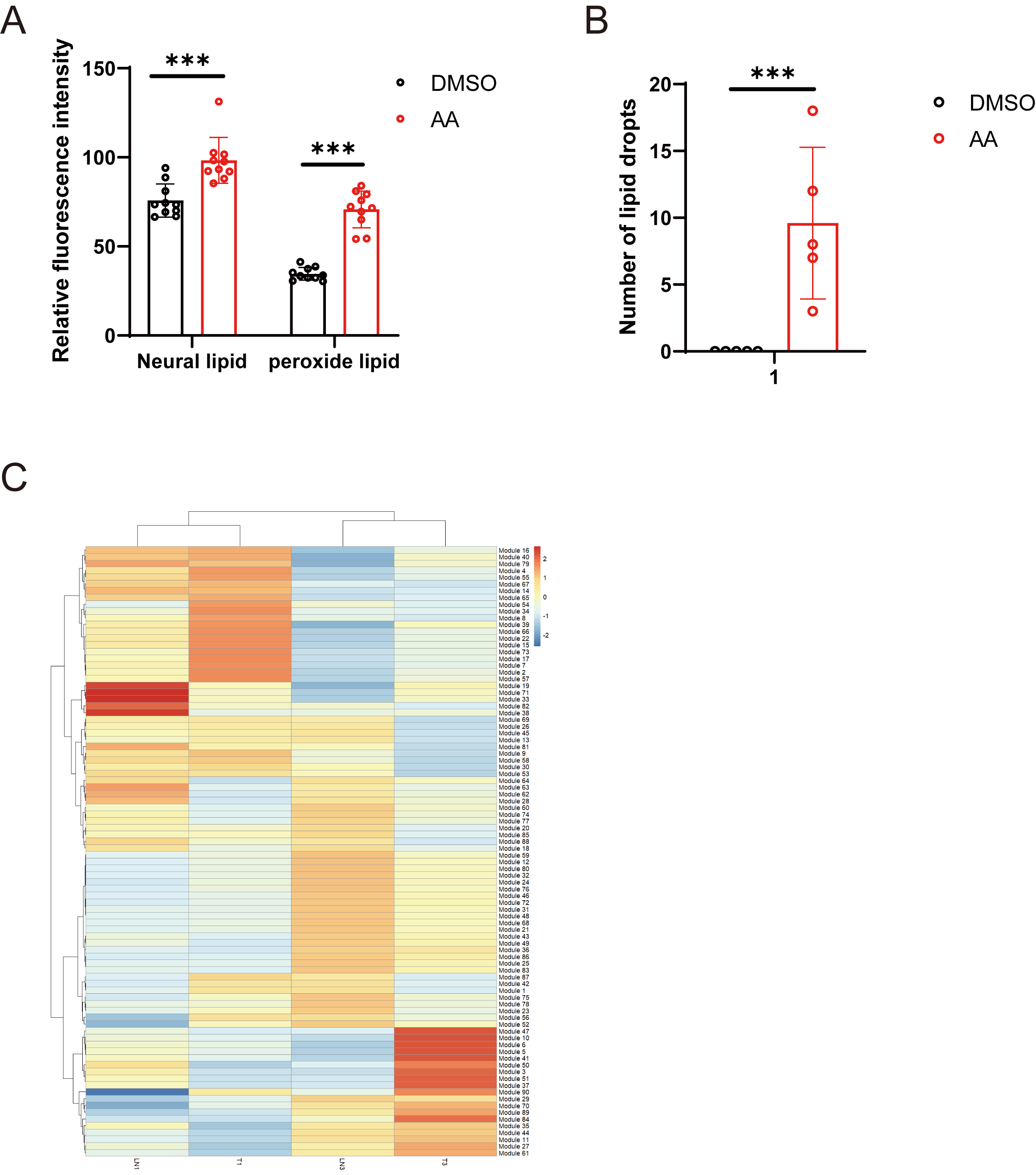
**Supplementary Figure 5. AA induced ferroptosis phenotype in GC cells.** A. The fluorescence intensity of neural and peroxide lipids in MKN45 cells stained by BODIPY 581/591; B. The number of lipid droplets in MKN45 cells with or without AA treatment. C. Expression changes of the modules generated by Monocle 3 analysis;


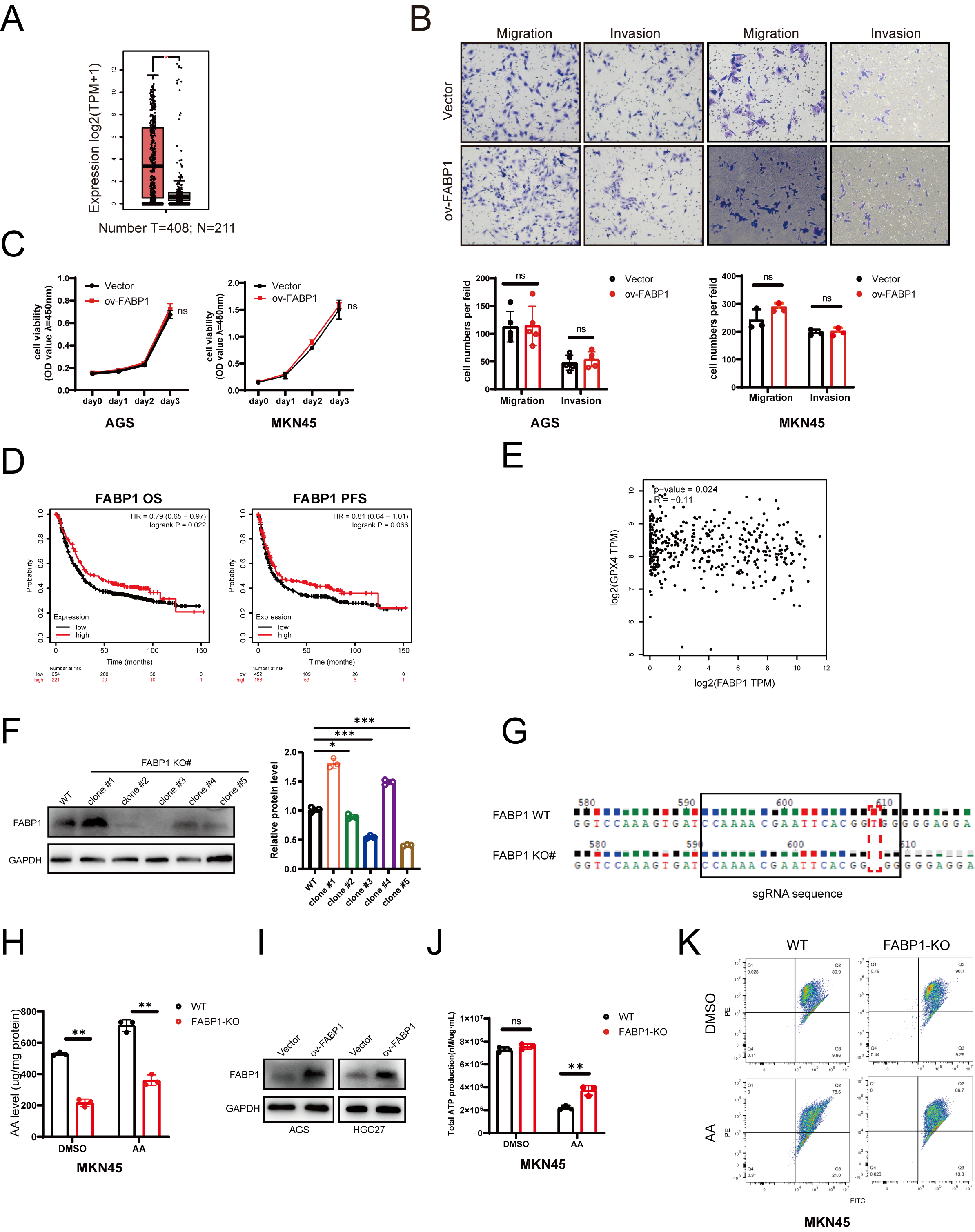


**Supplementary Figure 6. FABP1 mediated AA intake in GC cells.** A. FABP1 RNA levels in tumor and normal tissues from TCGA and GTEx database; B. Transwell analysis explored the effect of FABP1 overexpression on migration and invasion ability; C. CCK-8 analysis detected the proliferation ability with FABP1 overexpression; D. K-M plots showed the over-all survival (OS) and progression-free survival (PFS) in GC patients with FABP1 high or low expression from TCGA database; E. The correlation between FABP1 and GPX4 RNA levels in GC samples from TCGA; F. Western blotting results and band density analysis showed the knockout efficiency of FABP1 in different clones; G. Sanger sequencing detected the target fragment sequence of FABP1 knockout; H. ELISA analysis detected the Arachidonic acid level in GC cells on FABP1 knockout. I. Western blotting showed the FABP1 overexpression efficiency in GC cells. J. ATP levels in gastric cancer cells with FABP1 knockdown under AA treatment. K. Flow cytometry analysis of JC-1 in gastric cancer cells with FABP1 knockdown under AA treatment.
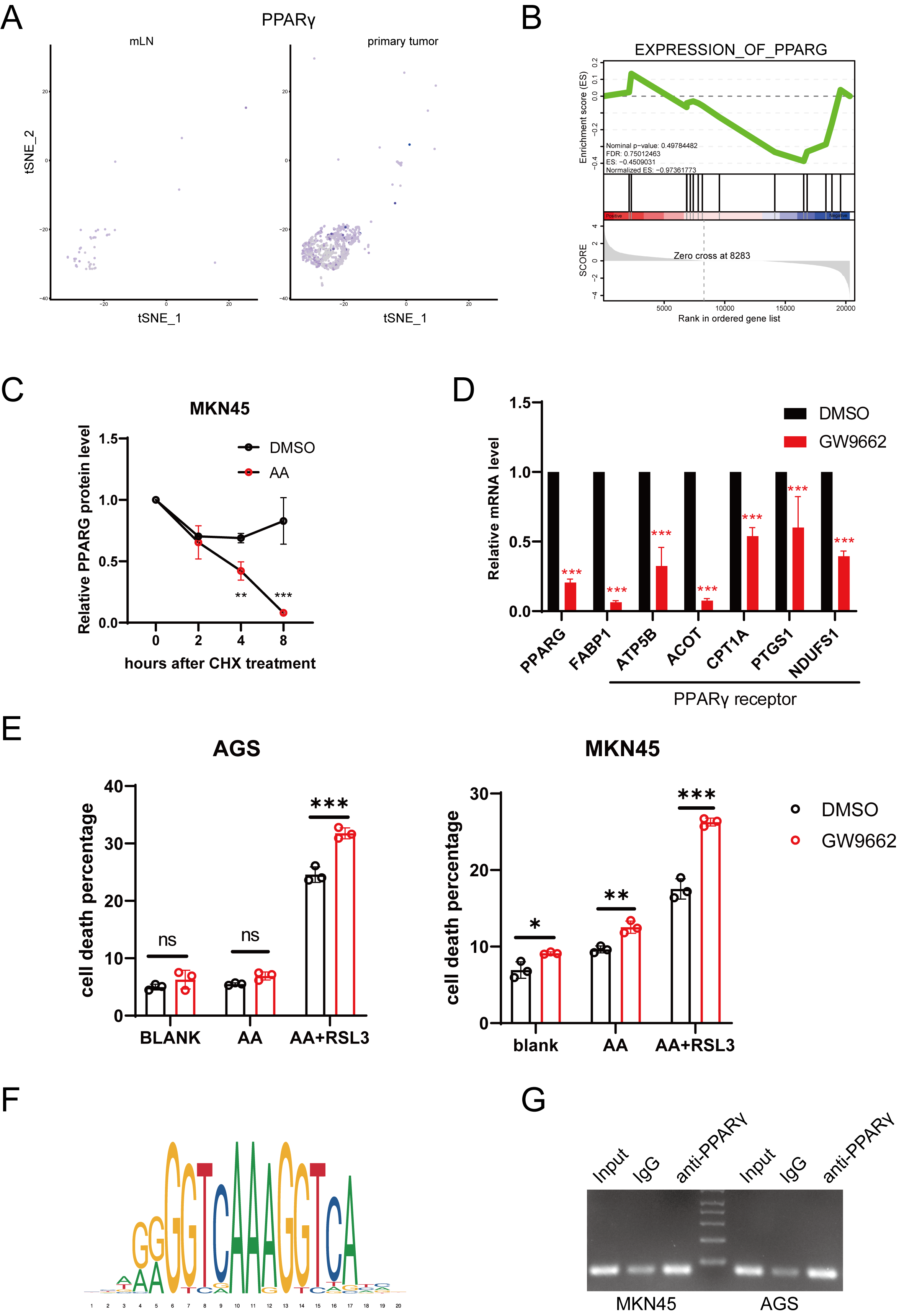


**Supplementary Figure 7. AA suppressed PPARG expression in GC cells.** A. Dot plot showed the PPARG expression levels in GC cells in sc-seq data; B. GSEA analysis of expression of PPARG pathway based on DEGs of GC cells in sc-seq; C. Gray value analysis of PPARG degradation on AA treatment; D. RT-qPCR revealed the RNA levels of target genes under GW9662 treatment; E. Flow cytometry analysis showed the cell death percentage of GC cells under AA(50uM), Rsl-3(2.5-5uM) and GW9662 treatment. F. The potential binding sequence of PPARG predicted by JASPR database; G. The southern blotting images of enriched DNA fragments from ChIP analysis.

**Supplementary Table**

**Supplementary Table S1. Sequences used in this study**

| **Primer sequence** |  |
| --- | --- |
| ACSL4-F | CATCCCTGGAGCAGATACTCT |
| ACSL4-R | TCACTTAGGATTTCCCTGGTCC |
| SCD1-F | GAGGCACCTACATTGGATGCT |
| SCD1-R | CGTAGACATAGGACCGCTCA |
| FABP4-F | ACTGGGCCAGGAATTTGACG |
| FABP4-R | CTCGTGGAAGTGACGCCTT |
| FABP1-F | GTGTCGGAAATCGTGCAGAAT |
| FABP1-R | GACTTTCTCCCCTGTCATTGTC |
| ELOVL5-F | TAACAGGAGTATGGGAAGGCA |
| ELOVL5-R | ACCAGAGGACACGGATAATCTT |
| FADS1-F | CCAACTGCTTCCGCAAAGAC |
| FADS1-R | GCTGGTGGTTGTACGGCATA |
| CD36-F | CTTTGGCTTAATGAGACTGGGAC |
| CD36-R | GCAACAAACATCACCACACCA |
| GAPDH-F | ACAACTTTGGTATCGTGGAAGG |
| GAPDH-R | GCCATCACGCCACAGTTTC |
| GCH1-F | GTGAGCATCACTTGGTTCCAT |
| GCH1-R | GTAAGGCGCTCCTGAACTTGT |
| PPARG-F | ACCAAAGTGCAATCAAAGTGGA |
| PPARG-R | ATGAGGGAGTTGGAAGGCTCT |
| CPT1A-F | TCCAGTTGGCTTATCGTGGTG |
| CPT1A-R | TCCAGAGTCCGATTGATTTTTGC |
| ATP5B-F | AAACAATTTGCTCCCATTCATGC |
| ATP5B-R | GACAACCTTGATACCAGTCACC |
| ACOT1-F | GAGGAAGGAGCGGTTTCCAC |
| ACOT1-R | AGGCGGCTTGCAGAAATAGTA |
| ACOX1-F | ACTCGCAGCCAGCGTTATG |
| ACOX1-R | AGGGTCAGCGATGCCAAAC |
| NDUFS1-F | TTAGCAAATCACCCATTGGACTG |
| NDUFS1-R | CCCCTCTAAAAATCGGCTCCTA |
| CYP4A10-F | CATGGCCGACTCCATTCGAT |
| CYP4A10-R | GCCTGGAGGTAGGTCCTGTA |
| PTGS1-F | CGCCAGTGAATCCCTGTTGTT |
| PTGS1-R | AAGGTGGCATTGACAAACTCC |
| p1FABP1-F | AAACCTTTGCTGTGCCCAT |
| p1FABP1-R | CCAGGTTCAAACATTAACTCCTG |
| p2FABP1-F | GGCCATAAAGGAATCAACAGC |
| p2FABP1-R | TGTAGGCTGTTTTATAGGGGGC |
| p3FABP1-F | TTTGAACCTGGCCATAAAGGA |
| p3FABP1-R | TTTTATAGGGGGCTCCCTTCC |
| **siRNA sequence** |  |
| Human-PPARγ-siRNA-1 | CUGACACCUAAGAAAUUUA dTdT |
| Human- PPARγ-siRNA-2 | CCCACUCCUUUGAUAUCAA dTdT |
| Human- PPARγ-siRNA-3 | GGAGAACAAUCAGAUUGAA dTdT |
| **sgRNA sequence** |  |
| FABP1-sg1 | GTGTCGGAAATCGTGCAGAA |
| FABP1-sg2 | GCCAAAACGAATTCACGGTGG |
| FABP1-sg3 | GAACTCAACGGCGACATAATC |

**Supplementary Table S2. Antibodies used in this study**

| Antibody | Application | Supplier |
| --- | --- | --- |
| FABP1 | WB 1:1000  IHC 1:250 | Abclonal (A11213) |
| PPARG | WB 1：1000  CoIP 1：100  ChIP 5μg | CST (C26H12) |
| GAPDH | WB 1:1000 | Bioss (10900R) |
| ME1 | WB 1:1000 | Abclonal (A3956) |
| RXR | WB 1:1000 | Abclonal (A19105) |
| SCD-1 | WB 1:1000 | Abclonal (A26246) |
| SOD2 | WB 1:1000 | Abclonal (A21805) |
| CK-18 | IHC 1:200 | Proteintech (10830-1-AP) |
| peroxidase-conjugated secondary antibody | - | Servicebio (G1214) |
| Rabbit IgG | ChIP 5μg | CST (#2729) |
