# Supplementary material for: Gastric cancer adapts high lipid microenvironment via suppressing PPARG-FABP1 axis after arriving in the lymph node
Source: Redox Biol. 2025 Jul 17;85:103759. doi: 10.1016/j.redox.2025.103759 (PMC12304710; doi:10.1016/j.redox.2025.103759)
Supplement: Multimedia component 3 [file mmc3.pdf]

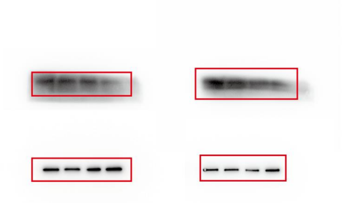
AGS MKN45

Figure 4E

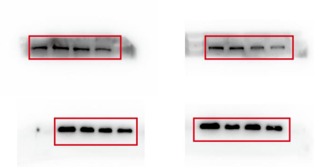
AGS MKN45

Figure 6B

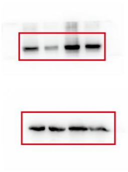
Figure 6E

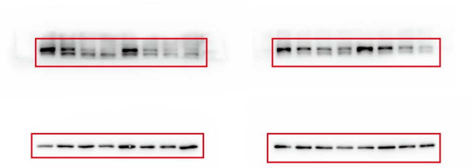
AGS MKN45

Figure 6D

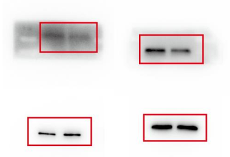
Figure 6G

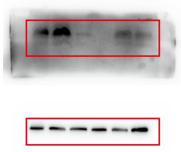
Supplementary Figure 6F

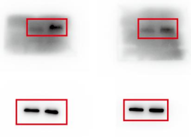
Supplementary Figure 6H

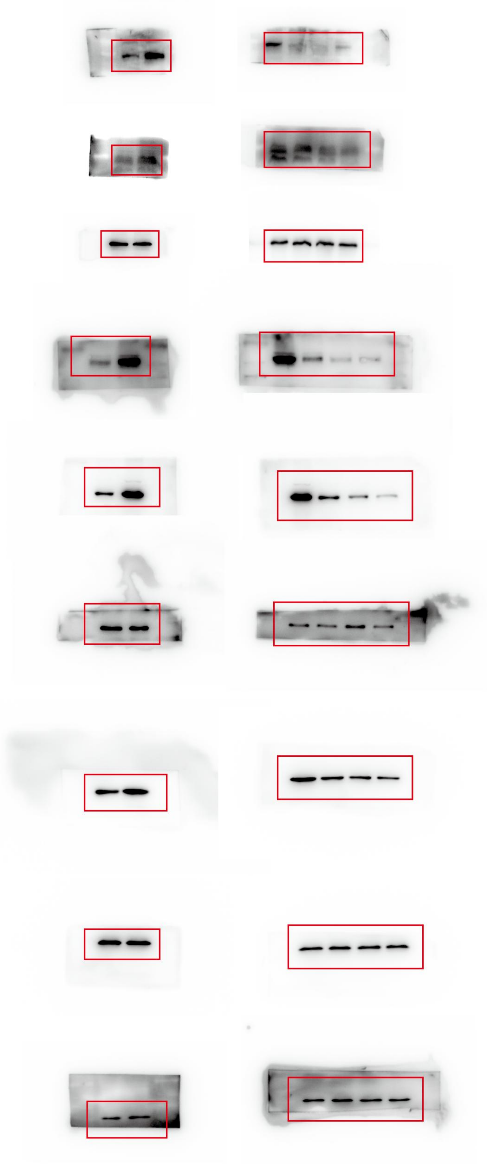
Figure 6F
